# Dark Traits, Harassment and Rape Myths Acceptances Among University Students

**DOI:** 10.1177/0306624X221139037

**Published:** 2022-12-07

**Authors:** Nicholas Longpré, Rowan E. Moreton, Emilie J. Snow, Fanni Kiszel, Molly A. Fitzsimons

**Affiliations:** 1 Edge Hill University, Ormskirk, UK; 2 University of Reading, Newbury, Berkshire, UK; 3 University of Roehampton, London, UK

**Keywords:** Dark Tetrad, Rape Myths, harassment, university students, sexual violence

## Abstract

The convergence of Machiavellianism, narcissism, psychopathy and sadism is known as the “Dark Tetrad.” Our understanding of the relationship between the Dark Tetrad, harassment and Rape Myths is limited. While men are more likely to blame victims of sexual violence, it is unclear how gender influences the ability to perceive harassment. The aim of the present study is to look at the relationship between dark traits, gender, Rape Myths and perception of harassment. A sample of *N* = 210 university students located in England & Wales were recruited on SONA and social media platforms. Student’s *t*-tests, Pearson’s correlations, and multiple linear regressions were conducted. Analyses revealed gender differences for both Rape Myths endorsement and perception of harassment. Furthermore, a relationship between the dark traits, Rape Myths and perception of harassment was founded. These results have several implications, including our ability to understand perpetrators’ characteristics, the impact of the Dark Tetrad on Rape Myths and perception of harassment, and our ability to develop effective prevention programs.

## Dark Triad to Dark Tetrad

In the past decade, a triplet of personality traits known as the “Dark Triad” has drawn attention (Paulhus & Williams, 2002). While personality traits associated to the Dark Triad are not necessarily pathological (i.e., subclinical), they increase predispositions toward cognitive distortions, utilitarian relationship, lack of perspective, lack of empathy, and callousness (Drislane & Patrick, 2017; Međedović & Petrović, 2015). The Dark Triad is composed of three facets: Machiavellianism, subclinical narcissism, and subclinical psychopathy. Machiavellianism involves manipulation to achieve one’s own goals, a lack of morality, a lack of empathy, and cynicism (Furnham et al., 2013). Narcissistic personality traits involve feelings of entitlement, superiority, grandiosity, and dominance (Morf & Rhodewalt, 2001). Finally, psychopathic personality traits involve callousness, a lack of empathy, and impulsivity (Paulhus, 2014). Recently, Patrick et al. (Drislane & Patrick, 2017; Evans & Tully, 2016; Patrick, 2010) have proposed a triarchic conceptualization of psychopathy that encompasses three distinct phenotypic constructs: disinhibition, boldness, and meanness. Disinhibition involves a failure to moderate actions and reactions based on the past or the future, and often leads to impulsive decisions; boldness can be considered as fearlessness, where individuals are often dominant and display little anxiousness; whilst finally, meanness is characterized by a lack of empathy, empowerment through cruelty, and a lack of social connectedness.

Recent research has suggested that everyday sadism should also be added to this set of traits (Paulhus, 2014). Everyday sadism represents a combination of behavioral and cognitive characteristics that are associated with the infliction of emotional and physical pain on others and a desire to control, punish and humiliate others in order to gain pleasure (Longpré, Guay et al., 2020). Studies have shown that everyday sadism and sexual sadism are strongly correlated (Longpré et al., 2019; Longpré, Guay et al., 2018). The conjunction of Machiavellianism, subclinical narcissism, subclinical psychopathy and everyday sadism is called the “Dark Tetrad” (Paulhus, 2014).

The scrutiny of the latent structure of the Dark Tetrad is part of an overarching debate about the nature of mental disorders with several studies suggesting that the majority of psychological disorders and personality disorders present a dimensional difference in the intensity of the disorder as opposed to a categorical difference in kind (i.e., Haslam et al., 2012). Although the four facets of the Dark Tetrad are overlapping, they have unique characteristics which make them distinct (Furnham et al., 2013). Studies have shown that the four facets of the Dark Tetrad are dimensional constructs that can be present among everyone, and across gender, but at different levels (Guay et al., 2007, 2018; Longpré, Guay et al., 2018; Plouffe et al., 2021). Recent studies have shown that the dark traits are good predictors of sexual violence (Beckett & Longpré, 2022), problematic sexual behaviors (Snow & Longpré, 2022), as well as stalking (Tachmetzidi Papoutsi & Longpré, 2022), which conceptually overlap with harassment (Longpré et al., 2022; Stefanska et al., 2022). The current manuscript aims to study the impact of the dark traits and gender on rape myths acceptance and perception of harassment.

## Sexual Harassment

Sexual harassment is defined as unwanted non-verbal, verbal, or physical behaviors with a sexual element, which aim to create an intimidating, humiliating, degrading, hostile, and/or offensive environment (Pina & Gannon, 2012; Pina et al., 2009). Sexual harassment is one of the most prevalent forms of sexual aggression, and extreme forms of harassment have been considered equivalent to rape (Pina et al., 2009). The #MeToo movement has shown that harassment and sexual violence are not isolated events and a need for more research is pressing (Snow & Longpré, 2022). Potter and Banyard (2011) reported that 38% of the women they interviewed had experienced sexual harassment within the workplace. Moreover, because of the high prevalence of harassment in the workplace and the army, studies of harassment have mostly focus on sociological causes, rather than on perpetrator characteristics (e.g., Lucero et al., 2006). Additionally, much of the past research into harassment has mainly focused on investigating the consequence on the victims, with less thought given to the relationship between the characteristics of the harassers and any underlying factors (O’Donohue et al., 1998).

Some studies have linked the Dark Tetrad with a higher inclination to engage in harassment and rape (Pavlović et al., 2019; Zeigler-Hill et al., 2016), with psychopathy having the strongest relationship (Brewer et al., 2021). However, much of the research on psychopathy and sexual violence is conducted on clinical or criminal samples, as opposed to the general population. Research exploring everyday sadism in relation to rape and harassment is also limited (Beckett & Longpré, 2022). Everyday sadism has been associated with online trolling and bullying (Buckels et al., 2019; Craker & March, 2016; Megarry, 2014; Sest & March, 2017), which could be linked to harassment by elements such as sending unwanted images, rape threats, sexualized bullying, and non-consensual sharing of intimate photos (Megarry, 2014). Subclinical narcissism has been associated with sexual violence, including sexual harassment, under specific circumstances such as after a sexual rejection (Jones & Olderbak, 2014), or when one felt, they were entitled to sexual contacts (Bushman et al., 2003). Jones and Olderbak (2014) have proposed that the relationship between subclinical narcissism and sexual violence is driven by ego-threats. However, the relationship between Machiavellianism and harassment remains elusive. Jonason and Webster (2012) have hypothesized that the relationship between Machiavellianism and harassment is relatively low, which could be explained by the moral flexibility associated to Machiavellianism (Jones & de Roos, 2017b). Furthermore, Machiavellianism has been negatively associated with short-term sexual behaviors (Jones & de Roos, 2017a). Despite this Brewer et al. (2021) did not find a relationship between Machiavellianism and harassment. However, their sample was composed of women only, which might have impacted the results. Nevertheless, the exploitive aspect of Machiavellianism has been associated with sexual aggression (McHoskey, 2001) and therefore some of it features could potentially lead to harassment.

## Rape Myths

Every 2 minutes a woman is raped in America (Buddie & Miller, 2002), and 2.5% of women in England and Wales said that they had been a victim of a sexual offense (including attempted offenses) in the previous 12 months (Home office, 2013). However, only one in five women who experienced an assault in the last year reported it to the authorities (Home office, 2013) and less than a third will eventually report it to the authorities (de Roos & Jones, 2022a). Many reasons have been used to explain the high number of sexual assaults that are not reported (de Roos & Jones, 2022a, 2022b). One explanation is the desire to avoid being subjected to victim-blaming, where stereotypes are used to minimize the assault as the victim probably “asked for it” (Buddie & Miller, 2001), or it was “not really a rape” (Payne et al., 1999). These victim-blaming explanations are called Rape Myths, which are defined as false beliefs about rape that are used to move the blame from the perpetrators to the victim (Suarez & Gadalla, 2010).

Studies have reported a higher level of acceptance of Rape Myths among men than women (Beckett & Longpré, 2022; Hayes et al., 2016; Snow & Longpré, 2022), which in itself has further been linked to a lesser likelihood of intervening (Banyard & Moynihan, 2011). Furthermore, men are more likely to blame victims of sexual violence and be suspicious of sexual abuse disclosures (Suarez & Gadalla, 2010). On the other hand, women are more likely to both intervene, and to believe victims (de Roos & Curtis, 2021; de Roos & Jones, 2022a). A possible explanation for this is that women can identify a broader range harassing behavior (Cortina & Berdahl, 2008), and are more susceptible than men to view situations as problematic (de Roos & Curtis, 2021; de Roos & Jones, 2022a, 2022b).

Research has found positive relationships between the dark traits, the acceptance of Rape Myths and sexual coercion among university students (Beckett & Longpré, 2022; Boland, 2018). Brewer et al. (2021) found that women who score higher on the dark traits, specifically psychopathy, may be more inclined to victim blaming. Furthermore, Watts et al. (2017) reported a link between psychopathy and rape myth acceptance, indicating both a link between psychopathy and accepting Rape Myths and that disinhibited and callous traits are better predictors than boldness. Everyday sadism has also been associated with sexual coercion among men, and positively linked to both rape myth acceptance and sexual assault (Boland, 2018; Koscielska et al., 2020; Russell & King, 2016). It has been suggested that Machiavellianism would be mostly associated with “She lied” subfactor (Paulhus & Williams, 2002), and not necessarily to Rape Myths as a whole (Boland, 2018). These results indicate that even in small dose, the dark traits have an influence on perception, attitudes, and behaviors.

## Sexual Harassment, Coercion, and Bystanders: The Effect of the dark traits

Studies have shown that individuals who engage in harassment are also likely to be more coercive, to endorse more Rape Myths and adversarial sexual attitudes, to be more authoritarian, and to be more aggressive (Begany & Milburn, 2002; Lucero et al., 2006). These results suggest substantial similarities among those identified as harassers and those identified as coercive. In a recent student’s survey, one-third of the participants reported that they have witnessed harassment or sexual violence during their time at university and 53% of bystanders mentioned that they intervened (Revolt Sexual Assault, 2018). Bystanders, individuals who are present when an event takes place but are not directly involved, play a key role in prevention of further sexual violence (Reid & Dundes, 2017).

Nonetheless, the level of bystanders’ intervention is not the same for everyone, and is modulated by individuals’ characteristics including gender, and personality traits. For example, men are less likely to intervene than women (de Roos & Jones, 2022a, 2022b; Reid & Dundes, 2017). Moreover, personality traits are highly correlated with perception, attitudes, and behaviors (Paulhus et al., 2018), which in turn will modulate bystanders’ interventions (Reid & Dundes, 2017). A recent study by Leone et al. (2020) suggested that empathy is an important factor in bystander intervention. Whilst Leone did not specifically measure the Dark Tetrad, a lack of empathy is a core component of the dark traits. Therefore, it can be suggested that individuals with more empathy would be more likely to intervene as bystanders (Boland, 2018; Leone et al., 2020). However, Rape Myths can be seen as a barrier for empathy; it can stop them from perceiving the benefits of intervening and preventing an unwanted sexual experience. Therefore, even if an individual had empathy, their endorsement of Rape Myths could mitigate their likelihood of intervening as a bystander.

The relationship between perception and bystanders’ intervention is also influenced by the ability to understand a situation from another’s perspective. Indeed, individuals scoring high on lack of perspective were less likely to view scenarios as containing harassment (Leone et al., 2020) or sexual violence (de Roos & Jones, 2022a). A lack of perspective taking has further been associated with victim-blaming (Gravelin et al., 2019), and can hinder the likelihood of seeing harassment as problematic (Leone et al., 2020). Furthermore, an association between the perception of harassment and positive image has also been previously found (e.g., Sims-Knight & White, 2018).

Individuals who present themselves in a socially desirable manner (i.e., those presenting higher levels of social desirability) are usually more inclined to project favorable images of themselves (Midsa Clinical Manual, 2011). Therefore, these individuals identify harassment situations as socially undesirable and problematic. In a recent study, Longpré, Guay et al. (2018) found that social desirability was associated with more perspective taking, a necessary skill when making a judgment about the social acceptability of a situation, in this case, harassment. Additionally, the relationship between perception and action is mediated by social potency, which is defined as social dominance or interpersonal power and a desire to make an impact on others (Tellegen, 2000). Negative social potency has been associated with the Dark Tetrad in a previous study (e.g., Craker & March, 2016). Individuals presenting negative social potency are more likely to enjoy inflicting psychological pain and distress, are less likely to intervene in problematic situations, and employ negative social influence (Foulkes et al., 2014).

## Aims

The #MeToo movement has stressed the need to understand why individuals engage in harassment, and more research is needed on perpetrators’ characteristics (Beckett & Longpré, 2022). In order to effectively develop prevention programs, it is important to study the nomological network of harassment to understand which individual’s characteristics will modulate perception of harassment, as well as understand which factors can increase the risk of engaging in harassment and sexual violence.

The aim of the present study is to look at the relationship between three of the dark traits (Machiavellianism, everyday sadism, and subclinical psychopathy [measured by the TriPM subscales meanness and disinhibition]), Rape Myths and perception of harassment. A problem with Qualtrics have affected the data collected on Narcissism and only partial information was saved. Because it was impossible to guarantee that the information recorded were representative of the trends, the authors have preferred not to use the Narcissism scale in the analyses.

Furthermore, the impact of gender will be studied. Finally, external correlates associated to the dark traits, Rape Myths and/or harassment will be measured: Positive Image, Social Potency and Lack of perspective. It is hypothesized that individuals that are higher on one or more of the dark traits will endorse more Rape Myths and will less likely perceive harassment in various contexts. Furthermore, it is hypothesized that men will endorse more Rape Myths and will perceive less harassment when compared to women.

## Methods

### Participants

The participants for this study (*N* = 210) were university students recruited on SONA (*n* = 193) and social media (*n* = 17). The mean age of participants was 20.87 (*SD* = 4.36; Range = 18–47). The majority of participants were women (*n* = 160; 76.2%), Caucasian (*n* = 108; 51.4%), heterosexual (*n* = 172; 81.9%), and single (*n* = 133, 63.3%). For more details, see Table 1.

**Table 1.** Demographics.

	Mean	*SD*
Age in years	20.87	4.36
	N	%
Gender		
Female	160	76.2
Male	50	23.8
Non-binary	0	0
Prefer not to say	0	0
Ethnicity		
White/Caucasian	108	51.4
Black/African/Caribbean/Black British	29	13.8
Asian/Asian British	35	16.7
Arab	6	2.9
Other Ethnic Group	23	11
Prefer not to say	9	4.3
Sexual orientation		
Heterosexual	172	81.9
Gay/Lesbian	8	3.8
Bisexual	20	9.5
Pansexual	3	1.4
Prefer not to say	7	3.3
Relationship status		
Single	133	63.3
In a relationship	73	34.8
Other	4	1.9

### Procedures

The project has received ethical approval by a university in England & Wales. Consent form, socio-demographic questions, scales, and debrief form were added in Qualtrics, a web-based tool that allows to create and distribute surveys. It also allowed for anonymity as respondents and researchers were never in contact. First, a pilot was conducted on social media. Participants did not receive compensation. Following the pilot, participants were recruited in one university through SONA system, a cloud-based participant management software that is allowing researchers to set up online studies, recruit participants, and manage credits or paid participation. Participation did not involve any financial compensation, but they were awarded 1.5 credits for completing the survey.

Participants were presented with a detailed consent form that gave an overview of the study, including a warning about its sensitive nature, as well as the necessary information. The consent form also ensured that they were aware of their rights and that their data would be anonymous. At the end of the survey, participants were provided with resources for victims of sexual violence, both on- or off-campus supports.

### Scales

For the purpose of this study eight scales were used. For each scale, the total score was used in the analyses.

#### Positive Image Scale

This social desirability scale measures a participant’s likelihood to respond in a socially desirable manner to questionnaires (Midsa Clinical Manual, 2011). A higher score on this scale indicates a higher likelihood to respond in a socially desirable manner. The scale is composed of nine items, scored on a 5-point Likert-style scale (1—Definitely false, 2—Possibly false, 3—Not sure, 4—Possibly true, 5—Definitely true). An example of an item is “I am always polite, even to people who are rude.” In the present study, the Cronbach’s alpha was .69.

#### Social Potency Scale

This scale measure social potency, which is defined as a desire for social dominance and interpersonal power, and a desire to impact others. People who are strong in this personality trait are described as forceful, decisive, fond of influencing others, and fond of leadership roles (Tellegen, 2000). Studies have shown that social potency predicts Internet trolling (Craker & March, 2016), which is related to sadistic traits (Megarry, 2014). A higher score on this scale indicates a higher likelihood to use social dominance and interpersonal power. The scale is composed of 10 items, scored on a 4-point Likert-style scale (1—Strongly Agree, 2—Agree, 3—Disagree, 4—Strongly Disagree). An example of an item is “I have a natural talent for influencing people.” In the present study, the Cronbach’s alpha was .79.

#### Lack of perspective

This scale measures the ability to take the *perspective* of others and the propensity to do so (Midsa Clinical Manual, 2011). A lack of perspective has also been linked to Rape Myths and victim blaming (Gravelin et al., 2019). A higher score on this scale indicates a higher likelihood to ignore the *perspective* of others. The scale is composed of eight items, scored on a 5-point Likert-style scale (1—Definitely false, 2—Possibly false, 3—Not sure, 4—Possibly true, 5—Definitely true). An example of an item is “I find it difficult to see things from the "other guy’s" point of view.” In the present study, the Cronbach’s alpha was .72.

#### Short Sadistic Impulse Scale

This scale measure sadistic inclinations and the propensity to be cruel with others (O’Meara et al., 2011). A lower score on this scale indicates higher sadistic tendencies. The scale is composed of 10 items, scored on a 4-point Likert-style scale (1—Strongly Agree, 2—Agree, 3—Disagree, 4—Strongly Disagree). An example of an item is “Hurting people would be exciting.” In the present study, the Cronbach’s alpha was .88.

#### Machiavellianism Personality Scale (MACH-IV)

This scale measure Machiavellianism, which is characterized by playfulness, the ability to delay gratification, and interpersonal antagonism (Christie & Geis, 1970). The MACH-IV reflects beliefs in manipulative tactics (Machiavellian tactics), a cynical attitude to human nature (Machiavellian views), and a pragmatic morality (Machiavellian morality) (Jones & Paulhus, 2009). A higher score on this scale indicates a higher likelihood to Machiavellian inclination. The scale is composed of 20 items, scored on a 5-point Likert-style scale (1—Strongly Agree, 2—Agree, 3—Neutral, 4—Disagree, 5—Strongly Disagree). An example of an item is “Anyone who completely trusts anyone else is asking for trouble.” In the present study, the Cronbach’s alpha was .60.

#### Triarchic psychopathy measure (TriPM)

The brief Triarchic scales measure boldness, meanness, and disinhibition components of psychopathy (Evans & Tully, 2016). For the purpose of the study, meanness and disinhibition were measured. The meanness scale is composed of 14 items, scored on a 4-point Likert-style scale (1—True, 2—Somewhat True, 3—Somewhat False, 4—False). An example of an item is “I do not care who I hurt to get what I want.” A higher score on this scale indicates a higher likelihood to be mean. The disinhibition scale is composed of 20 items, scored on a 4-point Likert-style scale (1—True, 2—Somewhat True, 3—Somewhat False, 4—False). An example of an item is “I have missed work without bothering to call in.” A lower score on this scale indicates a higher likelihood to be disinhibited. In the present study, the Cronbach’s alpha was .71 for the meanness scale, and .88 for the disinhibition scale.

#### Illinois Rape Myth Acceptance Scale (IRMA)

The 22-item IRMA scale measure perceptions toward rape-supportive cognitions and victim blaming attributions (Payne et al., 1999). The scale is composed of 22 items, scored on a 4-point Likert-style scale (1—Strongly Agree, 2—Agree, 3—Disagree, 4—Strongly Disagree), and 4 sub-scales: (1) She asked for it (six items), (2) He didn’t mean to (six items), (3) It wasn’t really rape (five items), and (4) She lied (five items). An example of an item is “If a girl is raped while she is drunk, she is at least somewhat responsible for letting things get out of hand.” A lower score on this scale indicates a higher endorsement of Rape Myths. In the present study, the Cronbach’s alpha was .93.

#### Perception Toward Harassment

This scale is an amended version of the MIDSA Harassment Scale (Midsa Clinical Manual, 2011). Instead of measuring behaviors, the amended scale measures perception. The scale is composed of 24 items, scored on a 4-point Likert-style scale (1—Strongly Agree, 2—Agree, 3—Neutral, 4—Disagree, 5—Strongly Disagree). An example of an item is “I think it’s acceptable to write nasty or humiliating things about someone in a public place.” A higher score on this scale indicates a better perception of harassment. In the present study, the Cronbach’s alpha was .94.

### Analyses

First, power analyses using G*Power software revealed that the sample size was sufficient to uncover large to medium effect size across analyses. Furthermore, distribution of each scale was tested prior to the analyses. Skewness and kurtosis values were ranging between −2 and +2, which supported the normal distribution assumption (George & Mallery, 2010). Finally, Levene’s tests for homogeneity of variances were conducted, and none were significant, therefore, parametric analyses were chosen over non-parametric.

First, Student’s *t*-tests were conducted to assess the impact of gender on the score of each scale. Then, Pearson’s correlations were conducted to investigate the relationships between the total score of each scale. Finally, two multiple linear regressions were performed to assess which variables predicted (1) The perception toward harassment and (2) The endorsement of Rape Myths. Analyses were conducted with Statistical Package for Social Sciences (SPSS) Version 26 (IBM, New York, USA).

## Results

First, Student’s *t*-tests were conducted to assess the impact of gender (Women/Men) on the score of each scale. Results are presented in Table 2. A significant difference on the IRMA Scale was found (*t(210)* = 2.88, *p* = .004), as men scored lower than women, indicating greater acceptance of Rape Myths. A breakdown of the IRMA revealed that men scored lower on subscale 1 [She asked for it] (*t(210)* = 3.40, *p* = .001), subscale 2 [He didn’t mean to] ((*t(210)* = 2.88, *p* = .004), and subscale 4 [She lied] (*t(210)* = 2.45, *p* = .015). Finally, a significant difference on the Perception Toward Harassment was also found (*t(210)* = 2.61, *p* = .010), as men scored lower than women.

**Table 2.** Independent *t*-Test Analysis Between Gender and Scales (Total Score).

	Women	Men	
	(*n* = 160)	*(n* = 50)	*t*
Positive Image Scale	28.66 (5.97)	27.68 (6.54)	0.99 *ns*
Lack of perspective	29.21 (3.94)	28.22 (4.41)	1.50 *ns*
Social Potency Scale	24.33 (2.41)	24.04 (2.36)	0.73 *ns*
Short Sadistic Impulse Scale	34.91 (4.27)	34.68 (3.83)	0.34 *ns*
Machiavellianism Personality Scale	59.16 (7.01)	61.08 (6.62)	0.98 *ns*
TriPM—Meanness	30.84 (4.37)	31.70 (3.92)	−1.25 *ns*
TriPM—Disinhibition	59.41 (7.36)	60.34 (7.26)	−0.78 *ns*
Illinois Rape Myth Acceptance Scale (IRMA)	77.41 (9.71)	72.72 (11.04)	2.88**
IRMA—She asked for it	21.64 (2.99)	19.90 (3.67)	3.40***
IRMA—He didn’t mean to	20.35 (3.25)	18.80 (3.54)	2.88**
IRMA—It wasn’t really rape	18.76 (2.08)	18.62 (2.12)	0.86 *ns*
IRMA—She lied	16.65 (3.07)	15.40 (3.43)	2.45*
Perception Toward Harassment	90.38 (8.42)	86.72 (9.42)	2.61**

* *p* < .05. **p < .01. ***p < .001.

Second, Pearson’s moment correlations were conducted. The full results are presented in Table 3. Machiavellianism was significantly correlated with Rape Myths (*r* = .28, *p* < .001), and its sub-components (correlations ranging from .22 to .27), but not correlated with Perception of Harassment. Everyday sadism was significantly correlated with Rape Myths (*r* = .31, *p* < .001), its sub-components (correlations ranging from .24 to .37), and with Perception Toward Harassment (*r* = .52, *p* < .001). Meanness was not correlated with Rape Myths and with Perception Toward Harassment. Disinhibition was significantly correlated with Rape Myths (*r* = .24, *p* < .001), three of the four sub-components (correlations ranging from .15 to .31), and with Perception Toward Harassment (*r* = .37, *p* < .001). Finally, significant correlations were found between Perception Toward Harassment and Rape Myths (*r* = .44, *p* < .001), and its sub-components (correlations ranging from .34 to .39).

**Table 3.** Correlations Between Scales (Total Score).

	1	2	3	4	5	6	7	8	9	10	11	12	13
Positive Image Scale (1)	-	.34***	−.30***	.17*	−.20**	−.16*	.35***	.04	.03	.02	.01	.11	.13
Lack of perspective (2)		-	−.26***	.11	.12	.03	.21**	.05	.06	.05	.03	.03	.20**
Social Potency Scale (3)			-	.08	.20**	.07	.06	.11	.12	.12	.11	.02	.03
Short Sadistic Impulse Scale (4)				-	.06	−.23	.39***	.31***	.24***	.24***	.37***	.25***	.52***
Machiavellianism Personality Scale (5)					-	.03	.11	.28***	.25***	.22***	.23***	.27***	.06
TriPM—Meanness (6)						-	−.22***	.08	.08	.04	.09	.07	.09
TriPM—Disinhibition (7)							-	.24***	.15*	.11	.28***	.31***	.37***
Illinois Rape Myth Acceptance Scale (IRMA) (8)								-	.92***	.87***	.76***	.86***	.44***
IRMA—She asked for it (9)									-	.75***	.64***	.71***	.39***
IRMA—He didn’t mean to (10)										-	.53***	.61***	.34***
IRMA—It wasn’t really rape (11)											-	.56***	.47***
IRMA—She lied (12)												-	.36***
Perception Toward Harassment (13)													-

* *p* < .05. **p < .01. ***p < .001.

Third, linear regressions were conducted to assess which variables predict Perception Toward Harassment. Full results are presented in Table 4. Results indicated that gender ([Men = 1], *b* = −0.18, *t(210)* = −3.01, *p* = .003), Everyday sadism (*b* = 0.45, *t(210)* = 7.05, *p* < .001), and Disinhibition (*b* = 0.21, *t(210)* = 3.04, *p* = .003) significantly predict Perception Toward Harassment. These variables explained a significant proportion of variance, *R*^2^ = 0.36, *F*(9) = 12.06, *p* < .001.

**Table 4.** Regression Analysis for Variables Predicting Perception of Harassment.

Variables	B	*b*	*t*
Gender (Men = 1)	−3.67	−0.18	−3.01**
Age	−0.01	−0.01	−0.02 *ns*
Positive Image Scale	−0.05	−0.04	−0.59 *ns*
Lack of perspective	0.25	0.11	1.78 *ns*
Social Potency Scale	0.05	0.01	0.23 *ns*
Short Sadistic Impulse Scale	0.94	0.45	7.05***
Machiavellianism Personality Scale	0.04	0.03	0.57 *ns*
TriPM—Meanness	0.15	0.07	1.19 *ns*
TriPM—Disinhibition	0.25	0.21	3.04**

*R*^2^ = 0.36 (*N* = 210, *p* < .001).

** *p* < .01. ***p < .001.

Finally, linear regressions were conducted to assess which variables predicted Rape Myths. Results are presented in Table 5. Results indicated that gender ([Men = 1], *b* = −0.24, *t(210)* = −3.67, *p* < .001), everyday sadism (*b* = 0.25, *t(210)* = 3.63, *p* < .001), and Machiavellianism (*b* = 0.28, *t(210)* = 4.22, *p* < .001) significantly predicted Rape Myths. These variables explained a significant proportion of variance, *R*^2^ = 0.25, *F*(9) = 7.15, *p* < .001.

**Table 5.** Regression Analysis for Variables Predicting Rape Myths.

Variables	B	*b*	*t*
Gender (Men = 1)	−5.62	−0.24	−3.67***
Age	−0.03	−0.01	−0.20 *ns*
Positive Image Scale	0.07	0.04	0.53 *ns*
Lack of perspective	−0.25	−0.10	−1.44 *ns*
Social Potency Scale	0.23	0.05	0.78 *ns*
Short Sadistic Impulse Scale	0.61	0.25	3.63***
Machiavellianism Personality Scale	0.41	0.28	4.22***
TriPM—Meanness	0.08	0.03	0.50 *ns*
TriPM—Disinhibition	0.19	0.13	1.81 *ns*

*R*^2^ = 0.25 (*N* = 210, *p* < .001).

*** *p* < .001.

## Discussion

### Overview of the Results

The aim of the present study was to look at the relationship between three of the dark traits, Rape Myths and perception of harassment. Furthermore, the impact of gender and external correlates were examined. Analyses revealed no difference between men and women on the dark traits. However, differences were found on the Perception Toward Harassment, on the IRMA score, and on the IRMA subscale “She asked for it,” “He didn’t mean to,” and “She lied.” Overall, these results indicate that women endorse fewer Rape Myths than men, and have a better perception of harassment. These results are in-line with our hypotheses.

Pearson’s r analyses revealed weak to moderate correlations between the dark traits, which is in line with the idea that while the facets of the Dark Tetrad are overlapping, they have unique characteristics which make them distinct (Paulhus & Williams, 2002). Correlations were also found between Rape Myths and perception of harassment, indicating that these two forms of sexual violence are related. Finally, correlations were found between the dark traits, Rape Myths and perception of harassment, which is also in-line with our hypotheses.

The first regression model revealed that being a man, being more sadistic, and being more disinhibited were associated with a lower perception of harassment. The second regression model revealed that being a man, being more sadistic, and being Machiavellian were associated with the endorsement of more Rape Myths. These results are partially supporting our hypotheses.

### Implications

Harassment and sexual violence are not isolated events and happen in several settings (Sims-Knight & White, 2018). The #MeToo movement has made it clear that women perceive harassment and sexual assault as intrusive, frightening, and harmful (Sims-Knight & White, 2018). Whilst sexually coercive behaviors have been under scrutiny, few researchers have explored the nomological network of harassment and its relationship with Rape Myths, the dark traits and its correlates. Recent studies have shown that sexual assault and harassment are not different in kind (Taxon) but are different levels on a dimension of sexual violence (Knight et al., 2018). These results are consistent with previous research that showed that subcategories of sexual violence are part of an Agonistic Continuum (Knight et al., 2013, 2018; Longpré et al., 2019; Longpré, Sims-Knight et al., 2020). Results steaming from our study have several implications, ranging from our ability to understand perpetrator characteristics, the impact of the dark traits on rape myths acceptance and the perception of harassment, to our ability to understand bystanders’ intervention and our ability to develop effective prevention programs that are tailored to empirical research.

#### Dark Traits and its correlates

The first aim of this study was to look at the relationship between the dark traits, external correlates, Rape Myths, and perception of harassment. Machiavellianism, everyday sadism, disinhibition, and a lack of perspective were associated with Rape Myths and/or the perception of harassment. However, regression analyses revealed that the relationship between these predictors is complex, and future research should further explore any moderation/mediation effects. For example, the absence of a relationship between meanness and sexual violence might be the result of shared variance with sadism (for more details, see Longpré, Guay et al., 2018). This shared variance is supported by recent factor analysis which revealed that some items on the TriPM-Meanness and the SSIS are loading on the same factor (Spank-Villar, 2021).

Overall, our results are in-line with previous research; higher levels of sadism are usually associated to more sexual violence (Longpré, Guay et al., 2018), disinhibition and a lack of perspective are associated to a general disregard of others’ limits (Longpré, Guay et al., 2018), whereas Machiavellianism is not associated to sexual violence (Jonason & Webster, 2012). Individuals high on Machiavellianism usually show low levels of impulsivity (Jones & Paulhus, 2009), and have a flexible morality (Jones & de Roos, 2017a), which are usually not associated with short-term sexual behaviors (Jones & de Roos, 2017a). This can explain why Machiavellianism was not a significant predictor of harassment in this study. However, this lack of relationships can also be explained, in part, by the overlapping variance between the dark traits. It was reported in previous study that some items measuring Machiavellianism and psychopathy overlapped (e.g., Muris et al., 2017). Nevertheless, individuals high in Machiavellianism are using more manipulation, whilst individuals high in psychopathy are usually using more coercion. This suggests that while some of these traits are similar, they may differ in terms of motivations and tactics used (Jones & Olderbak, 2014; Smith et al., 2018). Thus, some dark traits are less likely than others to lead to sexual violence because of the different underlying psychological process involved.

Furthermore, our results can explain, in part, why the level of bystander intervention is not the same for everyone and how individual characteristics can influence harassment and coercive behaviors. For example, if observers are perceiving similarities with the perpetrator, they will express more empathetic attitudes toward the perpetrator compared to the victim (de Roos & Curtis, 2021; de Roos & Jones, 2022a). Whilst prevention programs cannot ethically target individuals that score higher on the dark traits, our results can guide which aspects need to be covered by prevention programs to reduce sexual violence, such as others’ point of view (lack of perspective), emotional recognition, and egalitarian relationships. Although covering these elements may have a limited impact for those on the upper end of the Dark Tetrad spectrum, it should help to reduce harassment and coercive behaviors from individuals on the lower end of the Dark Tetrad spectrum and increase bystander intervention.

#### Gender

The second aim of this study was to look at the effect of gender. Research has shown that the causes of sexual violence are similar across gender (Knight et al., 2018; Schatzel-Murphy et al., 2009; Sims-Knight & White, 2018). However, the majority of sexual violence is committed by men, especially on the upper end of the spectrum of severity (e.g., rape and sexual homicides; Longpré et al., 2019). Our results partially support these findings. While men and women did not differ on the dark traits, being a man was one of the best predictors of harassment and Rape Myths in both regression models. Furthermore, significant differences were found between men and women on both rape myths endorsement (as well as on three of the four subscales), and on the perception of harassment.

These gender differences can be used to guide the development of sexual violence prevention programs. Men did not differ from women on the IRAM “It wasn’t really rape” subscale, indicating that men are, overall, able to identify sexual violence as well as women. However, men were unable to adequately recognize harassment, and minimized sexual assault by using external elements. Previous research has shown that men usually do not recognize sexual violence in absence of clear cues such as victims’ emotional distress, are more excited by images of submissive women than by images of smiling and cooperative women, minimize sexual assault and endorse more Rape Myths. These results are in-line with the sexual inhibition hypothesis (e.g., Knight et al., 2013). Based on these findings, effective prevention programs should focus on sex education with an emphasis on defining harassment, deconstructing victim-blaming by exploring how it can affect disclosure, and how to understand a trauma from someone else’s points of view (i.e., increase perspective taking skills). These kind of holistic prevention programs should help to reduce future coercive or harassing behaviors, but also increase bystander intervention.

### Limitations

This study is not without limitations. Firstly, the sample is predominantly composed of women, with only 23.8% of the sample being men. This difference in prevalence between men and women might have impacted part of our results and need to be interpreted accordingly. Despite this, our results revealed an impact of gender on the endorsement of Rape Myths and perceptions of harassment, which is consistent with the previous literature. Furthermore, gender was one of the best predictors of harassment and Rape Myths in both regression models. Future research should aim to include a better representation of men in their sample and should also explore possible differences between binary and non-binary identifying groups. Moving forward, increased gender diversity in future studies of this nature would be beneficial.

A second limitation steamed from the specific sample under scrutiny. Our analyses were conducted on a sample composed of university students, with a majority from the same university, which may have affected the generalizability of the data. Our results might not be applicable across all socio-economic statuses and ages. However, recent surveys have revealed that between 30% and 50% of university students have experienced sexual harassment or assault on campus (Bull & Rye, 2018; Stanton, 2014). Therefore, studying Rape Myths and harassment with a sample of university students is still appropriate. Furthermore, recent studies have shown that similar variables which were found to predict sexual coercion in offenders convicted of sexual assault (antisociality, callousness, emotional dysregulation, and sexualization) were found to predict harassment and sexual coercion, for both men and women in the general population (Knight et al., 2018; Longpré, Sims-Knight et al., 2020; Sims-Knight & White, 2018).

Finally, problems with Qualtrics have affected the quality of the data collected on Narcissism, and only partial information was saved. In order to avoid making conclusion on potentially unreliable data, as it was impossible to guarantee that the partial information recorded were representative of the trends, the authors have preferred not to use the narcissism scale in the analyses. Therefore, the relationship between narcissism, Rape Myths, and perception of harassment was not studied in the present paper. Moving forward, this elusive relationship (e.g., Jones & Olderbak, 2014) need to be scrutinized.

## Conclusion

In summary, analyses revealed a relationship between gender, Rape Myths, and perceptions of harassment, with Men endorsing more Rape Myths and perceiving less harassment than women. Furthermore, a relationship between the dark traits (Machiavellianism, everyday sadism, and subclinical psychopathy [meanness and disinhibition]), Rape Myths, and the perception of harassment was founded. However, not all facets were predictors of aversive sexual conducts, with everyday sadism and psychopathy-disinhibited appearing to be the best predictors.

Expanding our understanding on the risk factors that lead an individual to engage in harassment behaviors can further support the evolution of much needed intervention programs as well as providing indicators for an early recognition of individuals’ risk with regards to prevention. Studies have shown that there are substantial similarities among those identified as harassers and those identified as coercive and preventing harassment could therefore help to reduce coercive behaviors. Overall, the current study provides valuable information by adding to previous research as well as being a starting point for the development of future research. Future research should focus on developing effective prevention programs that are tailored to empirical research.
